# In vitro and in vivo activity of miR-92a–Locked Nucleic Acid (LNA)–Inhibitor against endometrial cancer

**DOI:** 10.1186/s12885-016-2867-z

**Published:** 2016-10-26

**Authors:** Anna Torres, Joanna Kozak, Agnieszka Korolczuk, Paulina Wdowiak, Ewa Domańska-Glonek, Ryszard Maciejewski, Kamil Torres

**Affiliations:** 1 Laboratory of Biostructure, Chair of Human Anatomy, Medical University of Lublin, Jaczewskiego 4, Lublin, 20-090 Poland; 2 Department of Clinical Pathomorphology, Medical University of Lublin, Jaczewskiego 8a, Lublin, 20-090 Poland; 3 Chair of Human Anatomy, Medical University of Lublin, Jaczewskiego 4, Lublin, 20-090 Poland

**Keywords:** miR-92a, LNA-inhibitor, Endometrial cancer, Mice xenograft, Proliferation, In vivo

## Abstract

**Background:**

Endometrial cancer is the most common cancer of the female reproductive tract.

Based on our previous studies we speculated that miR-92a exhibited pro-oncogenic properties in endometrial cancer, and therefore its inhibition could be used as a therapeutic measure in this disease. Therefore in the present study we aimed to investigate both in vitro and in vivo if inhibition of miR-92a in endometrial cancer would limit cancer cells proliferation.

**Methods:**

miR-92a expression was evaluated in four endometrial cancer cell lines using qPCR. Inhibition of miR-92a activity was obtained in endometrial cancer cell lines by a transient transfection of a custom designed Locked Nucleic Acid (LNA)-Inhibitor, developed to work both in vitro and in vivo. In vitro proliferation studies were performed using *xCELLigence* RTCA DP system. In vivo experiment was performed in Cby.Cg-Foxn1 < nu>/cmdb mice bearing endometrial cancer xenografts, which were intraperitoneally injected with nine dosages of 25 mg/kg of miR-205-LNA-inhibitor.

**Results:**

qPCR revealed increased expression of miR-92a in HEC-1-B, Ishikawa and AN3CA cells. LNA-i-miR-92a inhibited endometrial cancer growth in vitro. It was also demonstrated that systemic administration of LNA-i-miR-92a was feasible and exerted inhibitory effect on endometrial cancer xenograft growth in vivo with only mild toxic effects in treated animals, however the effect was observed until 12^th^ experimental day and the last three dosages did not maintain the attenuating effect with the acceleration of tumor growth observed at the end and after cessation of the intraperitoneal therapy.

**Conclusions:**

Taken together, these results indicate that intraperitoneal delivery of miR-92a-LNA-modified-inhibitor is feasible, devoid of significant toxicity and moderately inhibits endometrial cancer growth in vivo, and therefore warrants further studies investigating other routes of inhibitor delivery possibly in other animal models.

**Electronic supplementary material:**

The online version of this article (doi:10.1186/s12885-016-2867-z) contains supplementary material, which is available to authorized users.

## Background

Endometrial cancer (EC) is a common and histologically heterogeneous malignancy and although several molecular pathways were connected with its molecular basis the pathogenesis of this malignancy has not been fully elucidated [1]. Recently endometrial oncogenesis was connected with alterations of microRNAs expression [2]. And a number of studies including our own research reported microRNAs as promising diagnostic and prognostic factors in EC [3–5].

MiR-92a, a member of miR-17-92 cluster, was reported deregulated in several cancers being most commonly linked with gastrointestinal malignancies and with colorectal carcinoma in particular [6, 7]. To our best knowledge we were first to report its overexpression both in tissues and in plasma of EC patients [3].

Based on those results and findings presented by other authors indicating the possible role of miR-92a in PTEN/PI3K/Akt/mTOR, which is a canonical pathway investigated in EC we hypothesized that miR-92a could act as oncogene and promote carcinogenesis in endometrial epithelium [8, 9].

Therefore in this study we aimed to investigate both in vitro and in vivo if inhibition of miR-92a in EC would limit cancer cells proliferation. Aiming at evaluation of translational potential of miR-92a inhibition we chose Locked Nucleic Acid (LNA)–inhibitor to induce the knock–down of miR-92a. LNA–inhibitors were previously successfully applied in mice and non-human primates studies and proven to act after systemic delivery [10, 11]. The miR-92a-LNA-inhibitor used in the presented study was custom designed to work both in vitro and in vivo with the similar efficiency. To our knowledge this is the first report to investigate in vitro and in vivo effects of miR-92a inhibition in EC.

## Methods

### Tissues samples

Tissue samples of normal endometrium were obtained from 31 patients operated due to benign gynecological pathologies other than of endometrial origin. None of the females enrolled in the control groups had a history of cancer or endometrial pathology. Fresh tissue samples (NE *n* = 16) were collected during the surgery within 15 min from the uterus removal and immediately immersed in RNAlater (Thermo Fisher Scientific, Waltham, MA, USA). After 24-h incubation in RNAlater in 4 °C, tissues were stored in −80 °C until RNA extraction. FFPE tissues (*n* = 15) used for the study were fixed with 10 % formalin and stored for maximum 10 years. Healthy endometrium included both proliferative and secretory phase epithelium. There was no overlap between fresh and FFPE tissue samples Medical University of Lublin Ethical Committee approved the study design (# KE-0254/201/2008). Informed consent was obtained from each study participant.

### Cell lines

Endometrial carcinoma cell lines HEC-1-B, AN3CA and KLE were purchased from ATCC (Manassas, VA, USA) and Ishikawa was purchased from Sigma-Aldrich (St. Louis, MO, USA). Cells were obtained directly from the cell banks that perform cell line characterizations utilizing short tandem repeat profiling and passaged in the authors’ laboratory for fewer than 6 months after resuscitation. HEC-1-B cell line was maintained in MEM (Thermo Fisher Scientific, Waltham, MA, USA) supplemented with 10 % fetal bovine serum (FBS), Ishikawa cell line was cultured in MEM (Thermo Fisher Scientific, Waltham, MA, USA), supplemented with 5 % FBS, KLE was maintained in DMEM, and AN3CA was cultured using EMEM (Thermo Fisher Scientific, Waltham, MA, USA), supplemented with 10 % FBS. All cell lines were maintained with supplementation of 2 % penicillin/streptomycin and incubated in humidified chamber at 37 °C in 5 % CO_2_ atmosphere.

### miR-92a-LNA-inhibitor and scramble control

miR-92a-3p-LNA-inhibitor (LNA-i-miR-92a) and scramble control (LNA-i-miR-NC) had full phosphorothioate (PS) backbones, were designed and purchased from Exiqon (Vedbaek, Denmark). LNA-i-miR-92a was custom designed to work effectively in human cells in vitro and in mice xenograft of human EC and had a following sequence: CGGGACAAGTGCAAT. LNA-i-miR-NC sequence was as follows ACGTCTATACGCCCA. Oligonucleotides used for the in vitro study were FAM labeled. The oligonucleotides were HPLC and Na salt exchanged purity level and were delivered lyophilized. The systemic effects of miR-92a-LNA-inhibitor could be reliably evaluated in vivo, as sequences of human and murine miR-92a-3p are identical.

### Transfection

Oligonucleotides were incubated with Lipofectamine RNAiMAX (Thermo Fisher Scientific, Waltham, MA, USA) at concentration of 12 pmol after dilution in OptiMEM (Thermo Fisher Scientific, Waltham, MA, USA). The mixture was added to cells and seeded into 16-wells or 96-wells plate at a density of 2×10^4^ cells per well depending on experiment format. Successful transfection (>50 % of all cells) was confirmed by visual fluorescence microscopic analysis.

### Luciferase reporter experiments

In order to additionally evaluate transfection efficiency and assess, if the studied inhibitor had a functional impact within studied cell lines we co-transfected 1×10^6^ EC cells with 50nM miR-205-LNA-inhibitor or scramble control and 30 ng/μl of pLightSwitch_3′UTR reporter vector containing optimized target sequence complementary to the miR-205-5p (based on miRBase 16) cloned downstream of RenSP luciferase gene (Acitve Motif, Carlsbad, CA, US). To evoke luciferase reporter signal 24 h following the transfections 100 μg of LightSwitch Assay reagent (Acitve Motif, Carlsbad, CA, US) was added to wells and after 30 min of incubation in the room temperature the luminescence was measured using VictorX4 multimode plate reader (Perkin-Elmer, Waltham, MA, USA). The experiments were performed according to manufacture’s protocol. The empty pLightSwitch_3′UTR reporter vector served a positive transfection control and a negative control for microRNA signaling. We also used pLightSwitch_Random 3′UTR Control 1 as an additional negative control for microRNA signaling. All experiments were performed in triplicates and repeated tree times.

### RNA isolation

RNA isolation from fresh tissues stored in RNAlater (Thermo Fisher Scientific, Waltham, MA, USA) and cell lines was performed using mirVANA^TM^ miRNA Isolation Kit (Thermo Fisher Scientific, Waltham, MA, USA) according to manufacturer’s protocol. Forty milligrams of macrodissected tissue was used for each isolation. After extraction RNA underwent DNase treatment using Turbo DNAase Kit (Thermo Fisher Scientific, Waltham, MA, USA).

Before RNA isolation from FFPE tissues 10 μm sections containing at least 70 % of cancer cells were prepared by microdissection. RNA isolation from FFPE specimens was performed using RecoverAll™ Total Nucleic Acid Isolation Kit for FFPE Tissues (Thermo Fisher Scientific, Waltham, MA, USA) according to the protocol provided by the manufacturer. After extraction RNA underwent DNase treatment using Turbo DNAase Kit (Thermo Fisher Scientific, Waltham, MA, USA).

### RNA quality control

RNA integrity was checked using Bioanalyzer 2100 (Agilent Technologies, Santa Clara, CA, USA) and Agilent RNA Nano kit. RIN values of RNA ranged between 6 and 8.6. Only samples with RIN ≥ 6 were used in downstream applications. Concentration and purity of RNA were measured using spectrophotometry and Biophotometer with Hellma TrayCell (Eppendorf, Hamburg, Germany). 260/280 ratio of all RNA samples ranged between 1.8 and 2.2. All samples were stored at −80 °C.

### Reverse transcription and qPCR

#### Reverse transcription

Reverse transcription (RT) was performed using TaqMan® MicroRNA Reverse Transcription Kit and specific primers (Thermo Fisher Scientific, Waltham, MA, USA). RNA extracted from tissues was reverse transcribed in 7.5 μl reactions. Each RT reaction consisted of: 3.5 μL RT Master Mix (0.75 μL 10xRT Buffer, 0.075 μL 100 mM dNTPs, 0.5 μL Multiscribe Reverse Transcriptase, 0.095 μL RNase Inhibitor (20U/μL), 2.08 μL Nuclease-free water), 1.5 μL of specific starters and 5 ng RNA in 2.5 μL solution.

RT reactions were carried using following protocol: 16 °C for 30 min, followed by 42 °C for 30 min and 85 °C for 5 min. All RT reactions were carried out in triplicates in Mastercycler ep gradient S (Eppendorf, Hamburg, Germany) and stored in −20 °C.

#### qPCR

qPCR was performed using single tube TaqMan® MicroRNA Assays and TaqMan® 2 × Universal PCR Master Mix, No AmpErase® UNG (Thermo Fisher Scientific, Waltham, MA, USA) in 10 μl reactions. For miRNA expression analysis in tissues each qPCR reaction consisted of: 0.5 μL 20×TaqMan® MicroaRNA Assay (Thermo Fisher Scientific, Waltham, MA, USA), 4.5 μL RT product (dilution 1:15), 5 μL 2× TaqMan® 2× Universal PCR Master Mix (Thermo Fisher Scientific, Waltham, MA, USA).

All qPCR reactions were performed in duplicates in ViiA7 Real–Time PCR System (Thermo Fisher Scientific, Waltham, MA, USA) using protocol suggested by manufacturer. Positive and negative control reactions as well as inter–plate calibrator (IPC) reactions were carried out on each plate. Raw qPCR data were initially normalized with IPCs and adjusted for reaction efficiency. Efficiencies of primer/probe sets were determined by performing standard qPCR with the six-fold dilution of a pool of ten randomly chosen cDNA templates. Efficiencies for all amplicons were calculated using the equation E = 10^(−1/slope)^-1.

For relative quantification of miRNA expression data were normalized using geometric mean of expression of experimentally chosen, stable endogenous controls RNU48, RNU44, U75 and U6 which were previously experimentally validated [12].

### xCELLigence real-time cells proliferation analysis

*xCELLigence* RTCA DP instrument (ACEA Biosciences Inc., San Diego, CA, USA) was placed in the humidified incubator at 37 °C and 5 % CO_2_ atmosphere. Cell proliferation experiments were carried out using E-plates according to manufacturer protocol. Cells were seeded into E-16 plate at a density 2×10^4^ in 100ul per well and experiment was running for 96 h. Each experiment was performed in triplicate. Data was analyzed using RTCA software and Slope was calculated every 12 h.

### Cell proliferation assay

Proliferation was measured using Delfia cell proliferation kit (Perkin-Elmer, Waltham, MA, USA) according to manufacturer protocol. BrdU incorporation was measured by time–resolved fluorescence 48 h after transfection using VictorX4 multimode plate reader (Perkin-Elmer, Waltham, MA, USA). All experiments were performed in triplicates and repeated tree times.

### Animals and in vivo study design

In vivo study was conducted in 15 female Cby.Cg-Foxn1 < nu>/cmdb mice aged 6 to 8 weeks with the body mass between 16.3 and 20.2 g. The animals were purchased from Centre of Experimental Medicine, Medical University of Białystok. The animals were housed in the sterile conditions and were monitored every other day for weight, physical activity and signs of distress. Ethical Committee of Medical University of Białystok approved study design and experimental procedures (# 7/2012).

EC xenografts were induced by subcutaneous injection of 50 μl suspension of HEC-1-B cells in PBS (1×10^7^ cells) into interscapular area. Mice were randomly divided into three groups (five animals in each group) and were treated with miR-205-LNA-inhibitor, scramble control or PBS, respectively. MiR-92a-LNA-inhibitor and scramble control were administered intraperitoneally in the dose of 25 mg/kg three times per week. Injections were commenced on the day following the HEC-1B cells injection. The experiment was conducted for 32 days. After the last injection, which took place on the 20 day of the experiment the mice were left for observation and they were sacrificed by anesthesia performed with 3 % isofluran. 0.5 mL of blood was retrieved from the heart of each animal and was preserved in EDTA for blood count. Tissues were collected and half of each organ was preserved for RNA isolation in RNA later and −80 °C, and the other half for histology studies in buffered 10 % formalin, and processed to paraffin blocks. Four micrometre slides were cut on the microtome and stained with hematoxylin and eosin (H + E).

### In vivo imaging

Near IR fluorescence imaging of live animals was performed on the 15^th^ experimental day using Odyssey Infrared Imaging System (LI-COR Biotechnology, Lincoln, NE, USA). Twenty-four hours before imaging the animals were injected with the epidermal growth factor antibody – EGF-IRDye-800CW (LI-COR Biotechnology, Lincoln, NE, USA), which was delivered via tail vein in the dosage of 1 nmol/L per mice. The affinity of the antibody to HEC-1B cells was assessed in the in vitro experiment prior to in vivo imaging.

### Statistical analysis

qPCR data processing and analysis was performed using GenEx 5.3.4. (MultiD). After normalization data were log transformed before statistical analysis. Results of functional experiments are presented as means with standard deviation (SD) of three independent experiments performed in triplicates. Comparisons between two groups were performed using Student’s *t* test with or without Welch correction depending on the equality of variances tested Fisher test. For comparisons between dependent groups the paired *t*-test was utilized. For multiple comparisons of independent groups ANOVA test with Tuckey-Kramer post-hoc test was applied. Statistical tests were two-sided and statistical significance was determined by *p*-value of less that 0.05. Statistical analyses were performed using MedCalc Statistical Software version 14.12.0 (MedCalc Software bvba, Ostend, Belgium; http://www.medcalc.org; 2014).

## Results

### MiR–92a expression in endometrial cancer cells

**Fig. 1:**
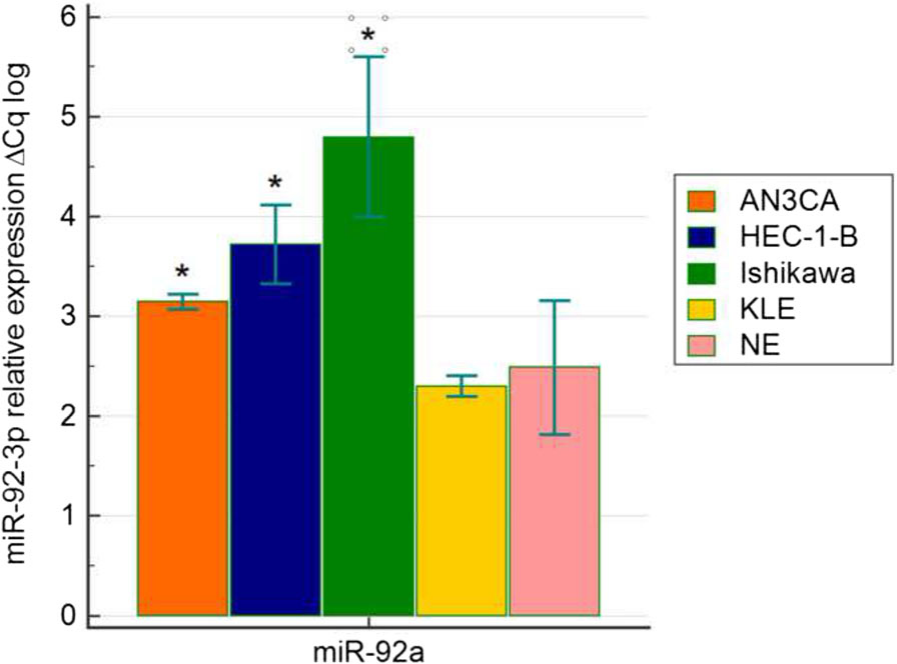
MiR–92a-3p expression in EC cell lines in comparison to normal endometrium (NE) tissues (*n* = 31); significant increase was observed in case of HEC-1-B (*p* = 0.013), Ishikawa (*p* = 0.008), AN3CA (*p* = 0.042)

Expression of miR–92a was significantly increased in HEC-1-B (*p* = 0.013), Ishikawa (*p* = 0.008) and AN3CA (*p* = 0.042) cells in comparison to normal endometrial samples (Fig. 1).

### Functional impact of miR-92a-LNA-inhibitor within studied cell lines

**Fig. 2:**
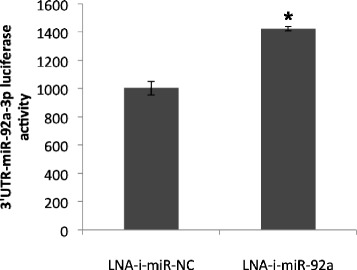
Specificity of LNA-i-miR-92a activity in HEC-1-B cells. The difference was statistically significant *p-*value = 0.023

In order to assess LNA-i-miR-92a activity in studied cell lines and verify its potential to knock-down miR-92a-3p pLightSwitch_3′UTR reporter vector containing optimized target sequence complementary to the miR-92a-3p was co-transfected with either miR-92a-LNA-inhibitor or scramble control. Luciferase activity was significantly increased 24 h after transfection proving that the inhibitor was efficient, biologically available and functional within the cells (Fig. 2).

### MiR-92a inhibits proliferation of EC cells in vitro

**Fig. 3:**
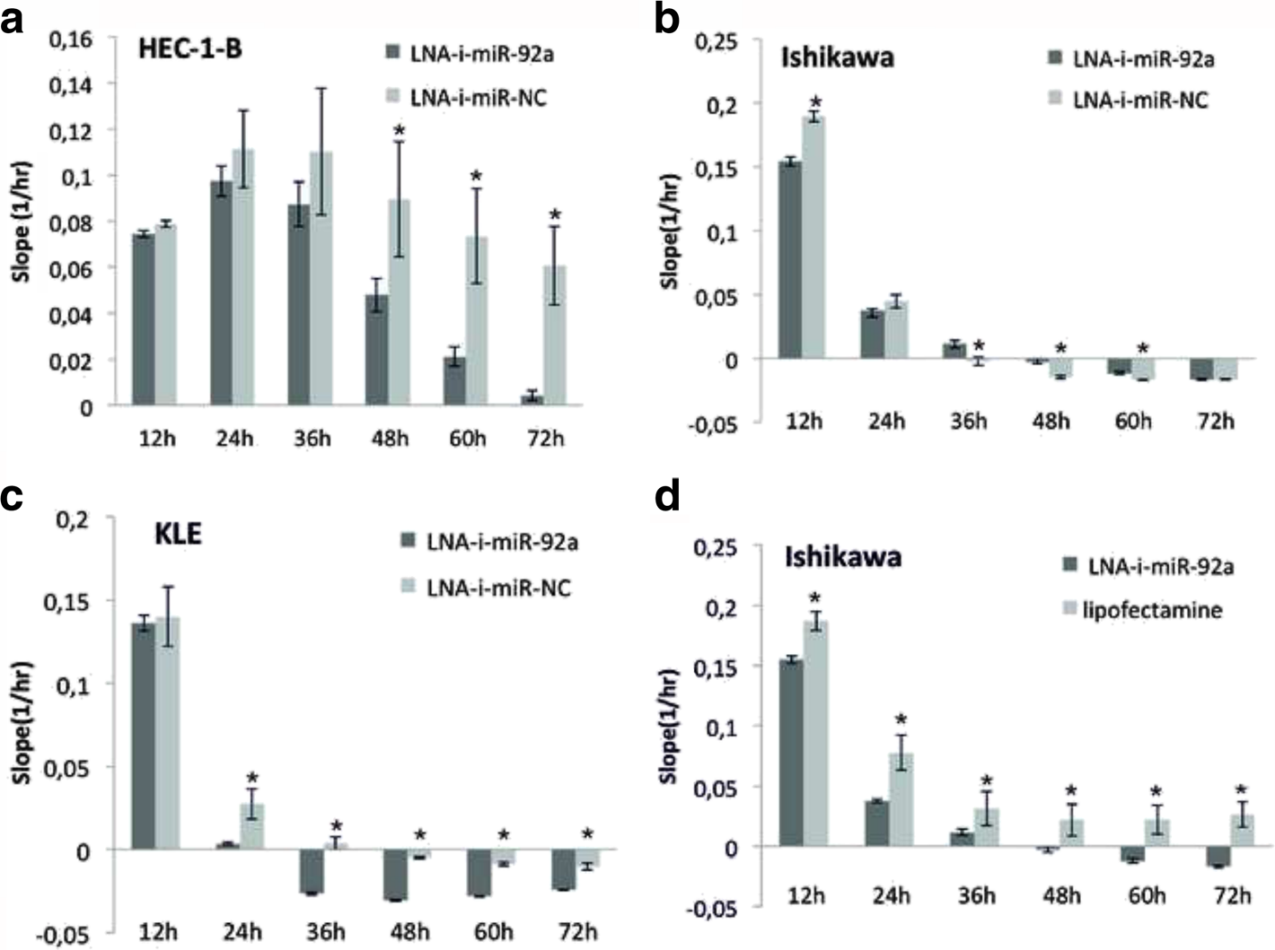
Antiproliferative effect induced by transient transfection of LNA-i-miR-92a in endometrial cancer cell lines compared to scramble control (LNA-i-miR-NC): **a** HEC-1-B, **b** Ishikawa, **c** KLE; **d** antiproliferative effect induced by transient transfection of LNA-i-miR-92a in Ishikawa cell lines compared to untreated control (lipofectamine). Averaged values ± SD from three independent experiments performed in triplicates are presented. * *p-*value <0.05

To assess anti-proliferative activity induced by LNA-i-miR-92a, we transfected HEC-1-B, Ishikawa and KLE cells and monitored cells proliferation for 72 h using *xCELL*igence technology. The xCELLigence system is a label-free cell-based assay system, which uses culture plates containing gold microelectrodes to non-invasively monitor the viability of cultured cells. The electrical impedance of the cell population in each well is measured by electrodes what provides quantitative real-time information about the cells’ condition. During the experiment the impedance value of each well was automatically monitored by the xCELLigence system for duration of 72 h. The rate of cell growth was determined by calculating the slope of the line between two given time points. Negative Slope means that cell index decrease with time and cells detach from wells bottom. Figure 3 presents the mean slope of three experiments performed in triplicates calculated at six time points between 12 and 72 h after transfection.

The transfection of LNA-i-miR-92a resulted in a significant inhibition of proliferation in HEC-1-B and KLE cell lines as compared to LNA-i-miR-NC. Stronger and earlier occurring effects were observed in KLE cells comparing to HEC-1-B cells. The proliferation rate of Ishikawa cells treated with LNA-i-miR-92a was lower, when compared to LNA-i-miR-NC at 12 h., however scramble control seemed to have more inhibitive effect during subsequent time points. Nevertheless rate of proliferation of Ishikawa cells was still significantly lower as compared to untreated control (Fig. 3a–d).

### In vivo effects of systemic administration of miR-92a-LNA-inhibitor

Effects of intraperitoneal administration of miR-92a-LNA-inhibitor were observed in experimental animals for 32 days. During this time animals obtained nine dosages of the inhibitor or LNA-i-miR-NC or PBS.

**Fig. 4:**
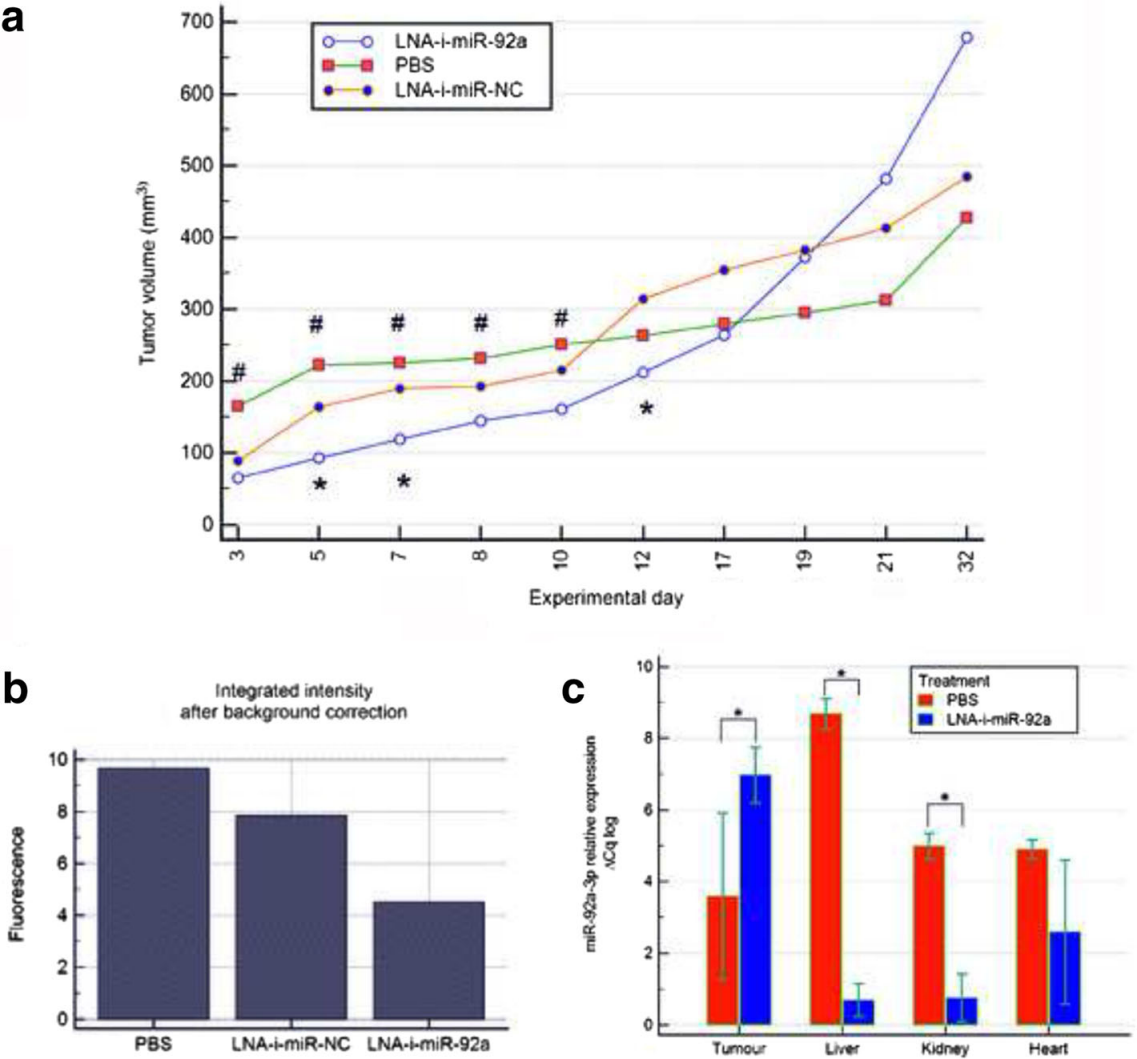
In vivo effects of systemic administration of miR-92a-LNA-inhibitor. **a** Subsequent measurements of tumor volume showed significant differences between miR-92a-LNA-inhibitor, scramble control (LNA-i-miR-NC) and not treated animals (PBS), * vs. scramble - *p-*value < 0.05, # vs. PBS – *p-*value < 0.05; **b** Because cancer cells over-express epidermal growth factor receptor, a non-invasive IR fluorescent-labeled probe for evaluating tumor size in vivo using Li-COR Odyssey Infrared Imaging Detection System was utilized; scanning tumor images revealed markedly reduced fluorescence intensity in the inhibitor group suggestive of an attenuating effect; in addition, no visible metastatic sites or extensive infiltration were revealed in either group – graph of representative animals; **c** MiR–92a-3p expression in mice tissues - significant difference was observed in case of tumor (*p* = 0.027), kidney (*p* < 0.0001), liver (*p* < 0.0001) and close to significant difference in heart (*p* = 0.063); bars represent Δ Cq log mean averaged values ± SD; *p-value < 0.05

Significant inhibition of tumor growth as compared to LNA-i-miR-NC was observed after second injection of miR-92a-LNA-inhibitor and it was observed until the 12th experimental day (after six dosages of miR-92a-LNA-inhibitor) (Fig. 4a).

In vivo imaging studies were performed on the 15^th^ day of experiment and were presented in Fig. 4b and Additional file 1. No visible metastatic sites or extensive infiltration were revealed in either group on scanning tumor with Li-COR Odyssey Infrared Imaging Detection System. Tumors retrieved from the animals at the end of the experiment were presented in the Additional file 2.

### MiR-92a-3p expression in mice tissues

At the end of the experiment miR-92a-3p expression was measured in tumors and as well as in vital organs. The results were presented in Fig. 4c. After treatment with miR-92a-LNA-inhibitor expression of miR-92a-3p was significantly decreased in liver (*p* < 0.0001), and kidneys (*p* < 0.0001), and close to significant decrease was noted in heart (*p* = 0.063). Conversely, increase of miR-92a-3p expression was found in tumor tissues (*p* = 0.014).

### Toxicity assessment of systemic administration of miR-92a-LNA-inhibitor

During the whole experimental period all animals tolerated procedures well and behaved similarly as assessed by activity level and food and water intake, which were normal. Body weights did not differ between three groups at the end of the experiment (Table 1). Measurements of organs retrieved from animals revealed significant increase of spleen and lungs in miR-92a-LNA-inhibitor group in comparison to PBS and scramble control. No differences were found in regards to weights of liver, kidneys, brain, heart, uterus and ovaries (Table 1). Complete blood counts were performed at the end of the experiment and no significant differences were observed in miR-92a-LNA-inhibitor treated animals in comparison to PBS or scramble control. There were no significant differences between scramble and PBS groups either (Table 2).

**Table 1:** Body weights and weights of organs collected from experimental animals

Organ weight (g)	LNA-i-miR-92a	LNA-i-miR-NC	PBS	*P* value		
	(1)	(2)	(3)	1 vs. 2	1 vs. 3	2 vs. 3
Mice weight	20.3	19.9	18.9	NS	NS	NS
Spleen	0.13	0.08	0.08	0.0005	0.004	
Liver	1.20	1.13	1.19	NS	NS	
Kidney	0.34	0.3	0.32	NS	NS	
Heart	0.11	0.12	0.13	NS	NS	
Lungs	0.16	0.14	0.14	0.01	0.04	
Brain	0.4	0.37	0.38	NS	NS	
Uterus	0.14	0.19	0.16	NS	NS	
Ovaries	0.04	0.04	0.04	NS	NS	

*NS* not significant

**Table 2:** Blood cell counts of experimental animals

Parameter	LNA-i-miR-92a	LNA-i-miR-NC	PBS	*P* value		
	(1)	(2)	(3)	1 vs. 2	1 vs. 3	2 vs. 3
Hb (g/dL)	11.7	13.2	12.7	NS	NS	NS
RBC (×10^6^/mm^3^)	7.5	8.3	7.85			
WBC (×10^3^/mm^3^)	1.5	1.8	1.7			
Ht (percent)	39.2	42.7	40.4			
PLT (×10^9^/L)	726	730	733			
MCV (xm^3^)	52.4	51.2	51.7			
MCH (pg/cell)	15.6	15.6	16.2			
MCHC (g/dL)	30.5	30.7	31.5			

*NS* not significant

### Histology studies

The microscopic examination of heart, spleen, pancreas, lung and brain did not show any significant lesions in any of the animals. There was also no sign of metastatic spread.

**Fig. 5:**
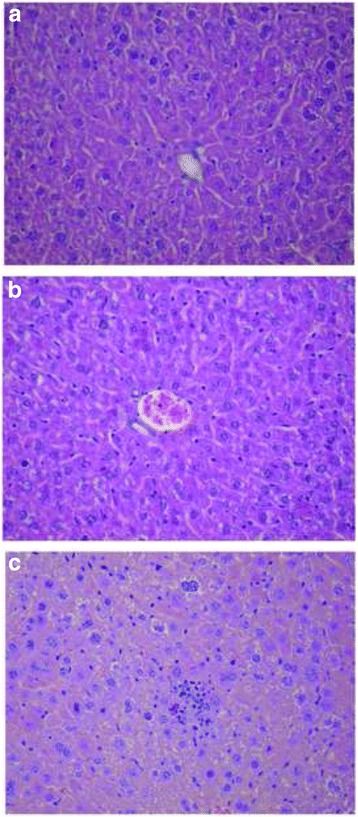
Histological changes encountered after treatment with miR-92a-LNA-inhibitor in mice liver; **a** PBS group - liver architecture within normal limits; **b** LNA-i-miR-NC group - liver architecture within normal limits; **c** LNA-i-miR-92a group - intralobular necrosis of hepatocytes with scattered mononuclear inflammatory cells and signs of hepatocytes regeneration with binucleated cells present; slides stained with H + E × 400

The microscopic examination of the liver samples from three miR-92a-LNA-inhibitor treated animals revealed changes in comparison to PBS and scramble control. The changes consisted of very small, discrete foci of necrosis of small groups of hepatocytes scattered within the lobules, that were accompanied by accumulation of mononuclear inflammatory cells, signs of liver cells regeneration including enlarged nuclei with prominent nucleoli, bi- or trinucleated hepatocytes and scattered mitotic figures. Single apoptotic cells, steatosis of single hepatocytes and focal proliferation of Kupffer cells were also observed (Fig. 5).

## Discussion

MiR-92a belongs to a highly conserved family, which arises from three paralog clusters miR-17-92, miR-106a-363, and miR-106b-25. Mature miR-92a can arise from an intronic miR-92a-1 located at chromosome 13q31-q32 and from miR-92a-2 encoded on chromosome Xq26.2. Deregulation of miR-92a was observed in various malignant tumors and its aberrant expression was related to promotion of tumor proliferation, invasion and metastasis as well as inhibition of cancer cells apoptosis [7]. However it was not connected with EC pathogenesis until our recent finding of its up-regulation in tissues and plasma of EC patients [3]. That observation was recently supported by the deep sequencing study of Lu et al., who found that miR-92a expression differed between Ishikawa and HEC-1-B lines, being increased in the latter. The authors also reported the inconsistent expression pattern of the miR-17-92 cluster between the two EC cell lines, with non-homologous miRNA species differentially regulated in those lines [13].

Based on those results we hypothesized that miR-92a exhibited pro-oncogenic properties in EC, and it could be a targeted in a gene–specific therapy. LNA-modified-oligonucleotides used in the presented study to knock-down miR-92a inactivate their target microRNAs by forming stable complexes with the targets, which are then sequestered within the cell [10]. Due to their chemical properties LNA-modified-oligonucleotides are stable in body fluids what facilitates their systemic delivery. Moreover, clinical trials indicated their clinical feasibility and safety in humans [14, 15].

Only few articles presented similar attempts of LNA-modified-oligonucleotides systemic delivery in human cancer models. Therefore, treatment schedule proposed in the presented study was developed based on consultations with the manufacturer and on the findings of Elmén et al., who reported that miR-122:LNA–antimiR duplexes were stable in mouse plasma over 96 h [10].

Increased expression of miR-92a in EC cell lines, as compared to healthy endometrial tissues was observed, what confirmed our previous results in human tissues [3]. The biological effect following transfection of miR-92a-LNA-inhibitor was confirmed in studied cell lines and efficient transfection conditions were verified by increased luciferase activity. Biological effect of miR-92a knock-down was inspected by proliferation studies, using xCELLigence real-time cells proliferation analysis, which allowed monitoring of time-resolved changes in EC proliferation.

LNA-i-miR-92a inhibited proliferation of HEC-1-B and KLE cell lines as compared to LNA-i-miR-NC, and the effect occurred earlier in KLE cells. Soon after transfection the proliferation rate of Ishikawa cells treated with LNA-i-miR-92a was lower when compared to LNA-i-miR-NC, however scramble control seemed to have more inhibitive effect during subsequent time points. Nevertheless rate of proliferation of Ishikawa cells was still significantly lower as compared to untreated control. The effect of non-targeting control was quite large in all our experiments in all endometrial cell lines, and therefore we speculate that this might have been due the non-specific effect of the LNA-technology.

Based on the in vitro results we speculated that systemic delivery of LNA-i-miR-92a could inhibit EC xenograft tumor growth in vivo. We found that administration of the inhibitor was feasible and was not connected with significant toxic effects. Similar minimal toxicities were seen in a study, which utilized miR-10b antagomirs in mice ovarian cancer xenograft model [16]. Contrarily, Di Martino et al. who evaluated effects of LNA-miR-221-inhibitor in the multiple myeloma mice xenograft did no observe any acute or chronic toxicities [17].

After intraperitoneal injection of 25 mg/kg LNA-i-miR-92a three times per week a significant decrease in tumor growth was observed until the 12^th^ experimental day. The last three dosages did not maintain the attenuating effect on tumor growth. We speculated that such phenomenon could be attributed to inefficient penetration of the inhibitor through the bulk of tumor tissue with intraperitoneal administration. Our speculations are supported by the study performed by Di Martino et al., who observed that intravenous administration of LNA-inhibitor in multiple myeloma mice xenograft resulted in more efficient inhibition of tumor growth in comparison to intraperitoneal administration [17].

Extended observation of the animals after discontinuation of treatment revealed marked acceleration of tumor growth in LNA-i-miR-92a treated animals, suggesting that the inhibitory effect of LNA-i-miR-92a needs to be sustained by regular administration of the inhibitor. Consistent with the final tumors measurements were miR-92a expression studies in mice tissues, which revealed its significantly higher levels in the tumor tissues from LNA-i-miR-92a treated animals. We suspect that such phenomenon could result from a rebound effect of increased miR-92a synthesis after the period of decreased availability during inhibitor treatment.

There are some limitations to our study, which we plan to address in the future. Firstly, we were not able to evaluate alternative root of inhibitor administration. Therefore it needs to be confirmed if intravenous administration could sustain the inhibitory effect on tumor growth. Secondly, the phenomenon of accelerated tumor growth after treatment discontinuation needs to be explained. As single miRNAs regulate several various pathways chances are that inhibition of mR-92a could adversely influence systemic or local defensive mechanisms in tumour bearing mice. Thirdly, it would be interesting to assess the dose dependent response to LNA-i-miR-92a and finally the investigations of in vivo response in xenograft derived from other endometrial cancer cells could be of interest, as it was proven in clinical studies that endometrial cancer is a heterogeneous entity in regards to response to anti-cancer treatment [18].

## Conclusions

In conclusion, our study showed that LNA-i-miR-92a was a potent inhibitor of EC growth in vitro. It also demonstrated that systemic administration of LNA-i-miR-92a was feasible and exerted moderate inhibitory effect on endometrial cancer xenograft growth in vivo with minimal toxic effects in treated animals. Taken together, these results indicate that systemic delivery of miR-92a-LNA-modified-inhibitor could be a promising treatment strategy for endometrial cancer and warrants further studies investigating other routes of inhibitor delivery, possibly in other animal models.

## Additional files

Additional file 1: Images of representative animals; scanning with Li-COR Odyssey Infrared Imaging Detection System images revealed markedly reduced fluorescence intensity within tumors in the inhibitor group suggestive of an attenuating effect; in addition, no visible metastatic sites or extensive infiltration were revealed in either group. (TIF 655 kb)
Additional file 2: Tumors retrieved from experimental animals: Nr. 1–5 PBS treated mice; Nr. 6–10 LNA-i-miR-NC treated mice; Nr. 11–15 LNA-i-miR-92a treated mice. (TIFF 828 kb)
