# Erratum to: In vitro and in vivo activity of miR-92a–Locked Nucleic Acid (LNA)–Inhibitor against endometrial cancer

**DOI:** 10.1186/s12885-016-2971-0

**Published:** 2016-12-07

**Authors:** Anna Torres, Joanna Kozak, Agnieszka Korolczuk, Paulina Wdowiak, Ewa Domańska-Glonek, Ryszard Maciejewski, Kamil Torres

**Affiliations:** 1 Laboratory of Biostructure, Chair of Human Anatomy, Medical University of Lublin, Jaczewskiego 4, Lublin, 20-090 Poland; 2 Department of Clinical Pathomorphology, Medical University of Lublin, Jaczewskiego 8a, Lublin, 20-090 Poland; 3 Chair of Human Anatomy, Medical University of Lublin, Jaczewskiego 4, Lublin, 20-090 Poland

## Erratum

After the publication of the article [1], the authors realised it contained an error with the Methods section. The following paragraph should be deleted from the original article:

### Cell proliferation assay

Proliferation was measured using Delfia cell proliferation kit (Perkin-Elmer, Waltham, MA, USA) according to manufacturer protocol. BrdU incorporation was measured by time–resolved fluorescence 48 h after transfection using VictorX4 multimode plate reader (Perkin-Elmer, Waltham, MA, USA). All experiments were performed in triplicates and repeated tree times.
