# Supplementary figures and images for: In vitro and in vivo activity of miR-92a–Locked Nucleic Acid (LNA)–Inhibitor against endometrial cancer
Source: BMC Cancer. 2016 Oct 26;16:822. doi: 10.1186/s12885-016-2867-z (PMC5080781; doi:10.1186/s12885-016-2867-z)

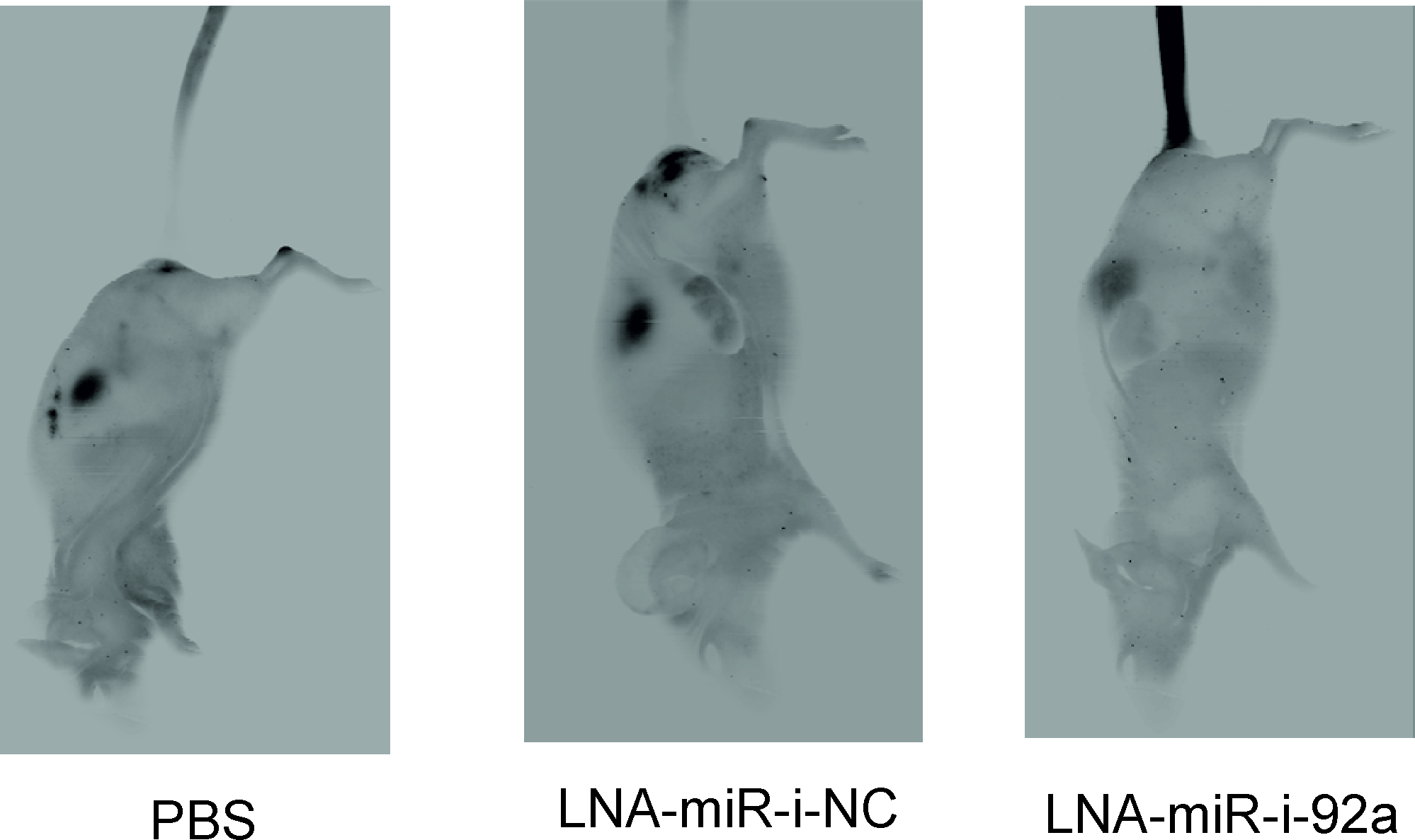

Supplement: Additional file 1: — Images of representative animals; scanning with Li-COR Odyssey Infrared Imaging Detection System images revealed markedly reduced fluorescence intensity within tumors in the inhibitor group suggestive of an attenuating effect; in addition, no visible metastatic sites or extensive infiltration were revealed in either group. (TIF 655 kb) [file 12885_2016_2867_MOESM1_ESM.tif]

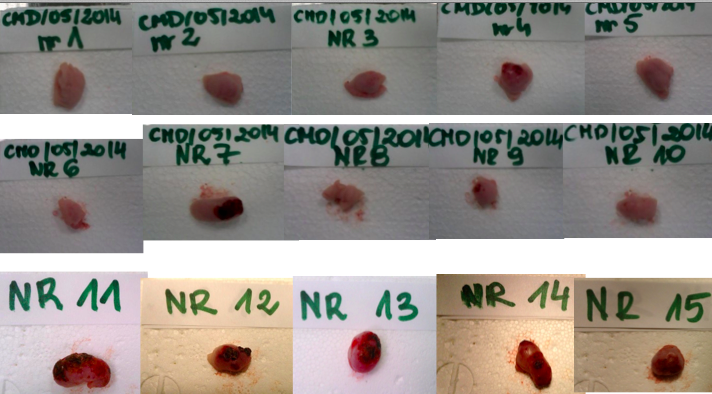

Supplement: Additional file 2: — Tumors retrieved from experimental animals: Nr. 1–5 PBS treated mice; Nr. 6–10 LNA-i-miR-NC treated mice; Nr. 11–15 LNA-i-miR-92a treated mice. (TIFF 828 kb) [file 12885_2016_2867_MOESM2_ESM.tiff]
